# A scientifically validated combination of garcinol, curcuminoids, and piperine for mild to moderate nonalcoholic steatohepatitis patients—results from a randomized, double-blind, placebo-controlled study

**DOI:** 10.3389/fnut.2023.1201186

**Published:** 2023-12-14

**Authors:** Muhammed Majeed, Kalyanam Nagabhushanam, Mazen Noureddin, Shaji Paulose, Chinmoy Barik, Santosh Saklecha, Lakshmi Mundkur

**Affiliations:** ^1^Sami-Sabinsa Group Limited, Bangalore, Karnataka, India; ^2^Sabinsa Corporation, East Windsor, NJ, United States; ^3^Houston Liver Institute, Houston Research Institute, Houston, TX, United States; ^4^JNM Hospital, Kalyani, West Bengal, India; ^5^Santosh Hospital, Bengaluru, Karnataka, India

**Keywords:** nonalcoholic steatohepatitis, curcuminoids, garcinol, piperine, Fibroscan, liver stiffness, FAST score

## Abstract

**Background:**

Garcinol is a naturally occurring compound from the fruit rind of the *Garcinia indica*, with antioxidant, anti-inflammatory, and anticancer properties. Curcuminoids are the active molecule from the rhizome of *Curcuma longa*, studied extensively for its health benefits as an anti-inflammatory and antioxidant activities. Non-alcoholic steatohepatitis (NASH) is the progressive form of nonalcoholic steatohepatitis characterized by liver fat and inflammation.

**Objective:**

To evaluate the clinical efficacy and safety of Garcinol, Curcuminoids and piperine (GCP) combination in patients with mild to moderate NASH in a randomized, double-blind, placebo-controlled study.

**Methods:**

The patients received one tablet (450 mg) of GCP containing garcinol-50 mg, curcuminoids −250 mg and piperine 5 mg or a placebo (450 mg of microcrystalline cellulose) twice daily for 90 days. Changes in circulating aspartate aminotransferase (AST), alanine transaminase (ALT) levels, liver stiffness measurement (LSM), and controlled attenuation parameter (CAP) using Fibroscan were compared from baseline to day 90. Anthropometric parameters, serum levels of lipids, Interleukin (IL-6), hsCRP, and adiponectin were estimated. Safety was evaluated by laboratory parameters and by monitoring adverse events.

**Results:**

Seventy-two patients were randomized and 63 (GCP = 32, Placebo = 31) completed the study. The mean age of the patients was 48.3 ± 8.7 years (36 males and 27 females). The mean reduction in AST (U/L) was 9.53 in GCP and 3.16 in placebo (*p* < 0.001) and that of ALT (U/L) was 13.47 in GCP and 7.43 in Placebo (*p* = 0.002). The liver stiffness and CAP scores showed a better reduction in GCP (0.56 kPa and 12.38 db/m) compared to placebo (0.064 kPa and 10.42 db/m) *p* < 0.05. Consequently, the noninvasive Fibroscan-AST (FAST) score reduction was also found to be significant in GCP compared to placebo. Additionally, body weight, lipid levels, hsCRP, and IL-6 in serum decreased, while adiponectin levels increased in GCP-supplemented participants compared to placebo. The combination of garcinol and curcuminoids was well tolerated with no significant changes in hematological and clinical laboratory parameters during the 90-day supplementation.

**Conclusion:**

Our results suggest that GCP could be a possible supplement for the management of NASH.

**Clinical trial registration**: https://clinicaltrials.gov/, identifier CTRI/2019/11/022147.

## 1 Introduction

Nonalcoholic fatty liver disease (NAFLD) is a chronic liver disease caused by the accumulation of excess liver fat in the absence of excessive alcohol consumption or any other liver injury. The rise in obesity and metabolic syndrome across the globe has increased the prevalence and severity of the disease, rendering it the most common indication for liver transplantation (1). About 10–20% of individuals with NAFLD progress to nonalcoholic steatohepatitis (NASH), an inflammatory progressive disease with a higher risk of liver cirrhosis and hepatocellular carcinoma (2).

The most alarming fact about NAFLD is its asymptomatic nature which may remain unnoticed for years (3). The incidence of NAFD is exponentially increasing, with a global prevalence of 30.05% in adults and an alarming increase in pediatric population (4, 5).

The prevalence of NAFLD is 75% in overweight people and 90% in obese individuals, suggesting obesity (6) to be a major risk factor. The disease is regarded as the hepatic manifestation of metabolic syndrome and is found to be associated with type-2 diabetes, high body-mass index, insulin resistance, polycystic ovarian syndrome, hyperlipidemia, and old age. Although drug discovery for NAFLD is a priority, no drug has been approved by the United States Food and Drug Administration or the European Medicines Agency till today (7, 8).

**Figure 1:**
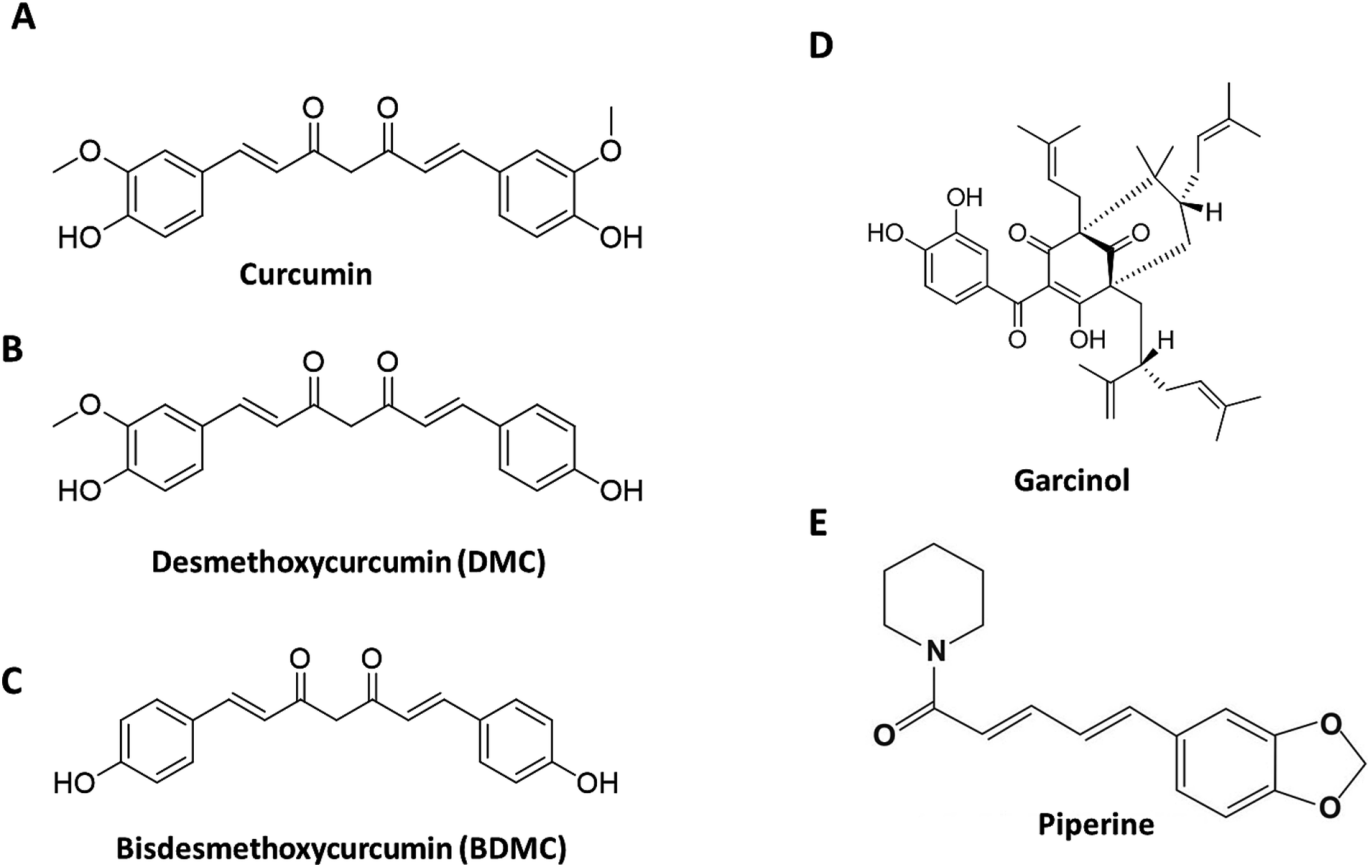
Structure of the active phytochemicals in the formulation. The structures of the Cucuminoids (A–C), Garcinol (D), and Piperine (E) present in the formulation.

The most effective treatment for fatty liver and NASH is lifestyle changes like exercise, caloric restriction, and nutrition. Vitamin E, pioglitazone, and new diabetic medications are used to treat NASH and its progressive conditions. Natural extracts and herbal products offer an alternate treatment for NAFLD. Curcuminoids refer to the three natural molecules: Curcumin (75–81%), Demethoxycurcumin (15–19%), and Bisdemethoxycurcumin (2.2–6.5%), present in the Curcumin C3 complex, the commercial extract from *Curcuma longa* rhizomes (turmeric). Garcinol is a polyisoprenylated benzophenone, extracted from the fruit rinds of *Garcinia indica* (Figure 1).

Curcuminoids were suggested to be a promising treatment for NAFLD in a meta-analysis of randomized clinical trials with a significant reduction in ALT, AST, and NAFLD severity (9, 10). Curcumin C3 complex at a dose of 500 mg with 5 mg piperine was reported to reduce NAFLD severity and decreased the serum levels of tumor necrosis factor, and monocyte chemoattractant protein in NAFLD patients (11). In another trial, Curcumin C3 complex at the same dose was reported to reduce ALT, AST, and lipid levels (12). Garcinol has been evaluated as an effective supplement for weight loss but has not been studied for NAFLD (13, 14). We have earlier reported that the combination of curcuminoids and garcinol was significantly more effective than the individual treatment in controlling the progression of liver steatosis to inflammation and fibrosis in the Stelic animal model (STAM™) model of NASH (15). In the present study, we explored the clinical efficacy of the combination of garcinol and curcuminoids with piperine in patients with mild to moderate NASH without liver cirrhosis. We used piperine as a bioenhancer in the present study, as the bioavailability of curcumin is known to increase in the presence of piperine (16). Bioenhancers are chemical entities, which promote and augment the bioavailability of natural molecules and drugs without showing any significant effect on their own. Piperine has been shown to increase the bioavailability of several natural products and drugs including curcumin (16–21). The dose of 250 mg of curcuminoids and 50 mg of 20% Garcinol, was derived from the preclinical study reported earlier (15).

## 2 Materials and methods

### 2.1 Materials

The test material was a combination of 20% garcinol from *Garcinia indica* (Livinol^®^—50 mg), 95% curcuminoids from *Curcuma longa* (Curcumin C3 Complex^®^—250 mg), and 95% piperine from *Piper nigrum* (BioPerine^®^—5 mg) as a bioavailability enhancer, referred to as GCP. This blend was formulated into a tablet (450 mg). The placebo tablet was formulated using microcrystalline cellulose (450 mg). The product was provided by Sami-Sabinsa Group Limited (LivLonga^®^). The details of the formulation are given in Supplementary methods section.

Curcumin C3 Complex was extracted from the dried rhizomes of *Curcuma longa*. In nature, the three curcuminoids exist together with Curcumin being the most abundant. The Curcumin C3 Complex used in the present study is composed of curcumin (75–81%), demethoxycurcumin (15–19%), and bisdemethoxycurcumin (2.2–6.5%). The product was standardized to contain not less than 95% w/w curcuminoids, with curcumin (80.22%), demethoxycurcumin (17.1%), and bisdemethoxycurcumin (2.68%) as per US pharmacopeial specifications.

Garcinol was extracted from the dried fruit rind of *Garcinia indica,* concentrated, and crystallized to get the pale-yellow needle-like crystals of garcinol with 75–80% purity. This 75–80% w/w of garcinol was diluted using microcrystalline cellulose to get 20% garcinol, containing around 5–7% of plant material, contributing to its stability at room temperature. Piperine was extracted from black pepper fruits (*Piper nigrum*) and standardized to contain 95% piperine. The HPLC chromatograms of Garcinol, Curcumin C3 Complex and Piperine are included in Supplementary Figures S1–S3.

GCP was formulated with garcinol (50 mg), curcuminoids (250 mg), and piperine (5 mg).

### 2.2 Analysis of GCP

**Figure 2:**
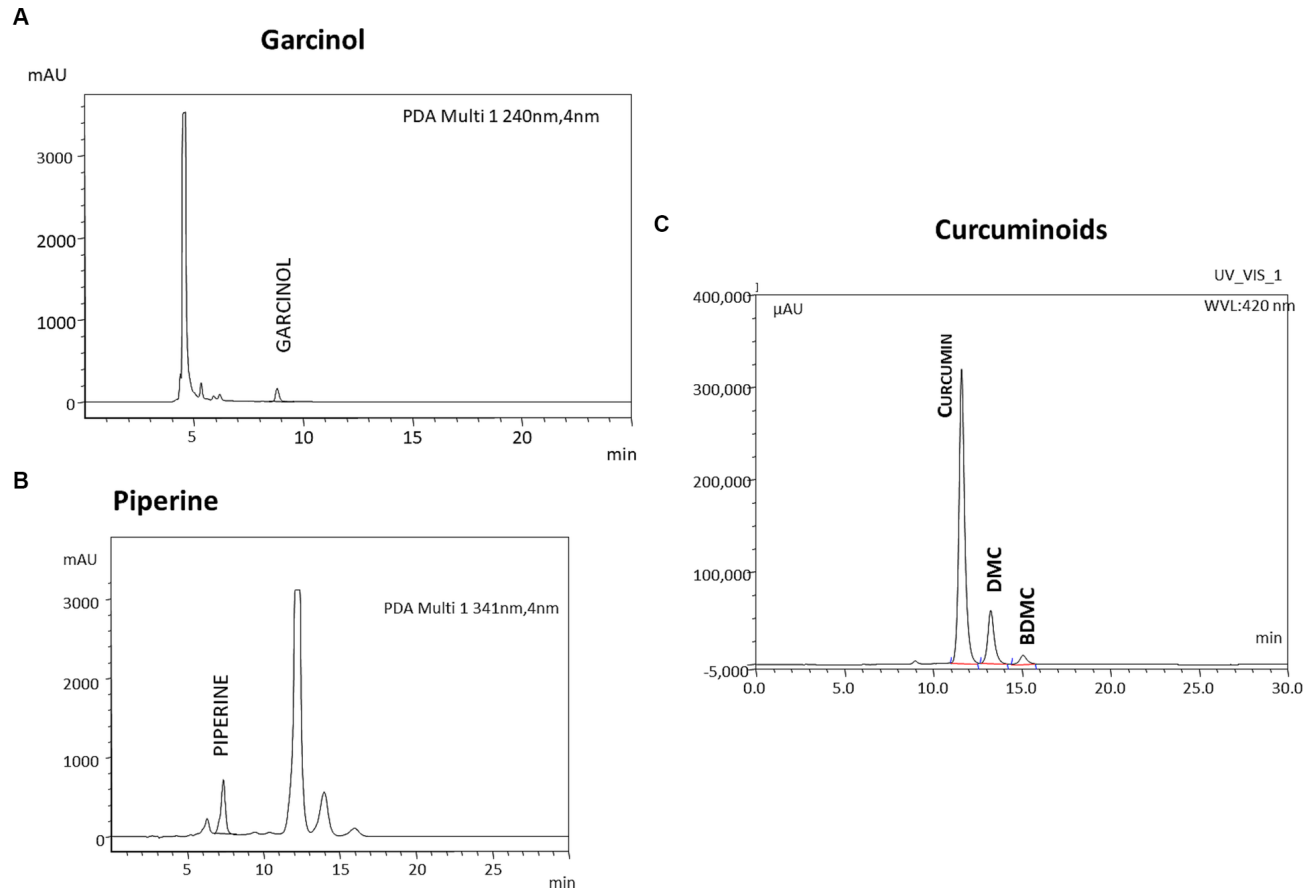
Representative HPLC profile of GCP. The formulation GCP was analyzed for the individual components using HPLC. (A) Garcinol is analyzed by reverse phase HPLC using Acetonitrile as mobile Phase and UV detector at 240 nm. (C) Curcuminoids (detected at 420 nm) and piperine (detected at 341 nm) were analyzed by reverse phase HPLC using Tetrahydrofuran and 0.1% Citric acid in the ratio of 40:60 (v: v) as (B) mobile phase.

Garcinol was again estimated by HPLC using a C18 ODS column, 4.6 mm (diameter) × 250 mm (length) with particle size 5 μ. The mobile phase consisted of 100% acetonitrile at a flow rate of 0.7 mL/min. A Photodiode array detector was used to detect garcinol at 240 nm. The additional early eluting peaks seen in the chromatogram are due to curcuminoids and piperine (Figure 2A).

Curcuminoids and Piperine were analyzed by HPLC using a C18 ODS column, 4.6 mm (diameter) × 250 mm (length) with particle size 5 μ. The mobile phase consisted of tetrahydrofuran with 0.1% w/v citric acid solution in the ratio 40:60 at a flow rate of 1 mL/min. A Photodiode array detector was used to detect curcuminoids at 420 nm (Figure 2B) and piperine at 341 nm (Figure 2C). and piperine at 341 nm using a UV detector Additional late eluting peaks seen in the chromatogram of piperine are due to curcuminoids.

### 2.3 Study design and ethics

A randomized, double-blind, placebo-controlled study was conducted from October 2020 to November 2021 in India. The study was designed at four centers; however, executed only at two sites, JNM Hospital, Kalyani, West Bengal, and Santosh Hospital, Bangalore, as the other two centers could not recruit any patients. All study documents were reviewed and approved by the institutional ethics committee of MS Ramiah Medical College and Hospital as per the study protocol number CPL/79/G&C_NASH/JUN/19 on 24 December, Dr. Satyprakash’s Center for Digestive and liver diseases, 11 October 2019, College of Medicine and JNM Hospital, 22 June 2020 and Santosh Hospital, 21 January 2021. The study was conducted following Good Clinical Practice as required by the International Conference on Harmonization. The trial was registered prospectively with the Clinical Trial Registry of India (CTRI) with the registration number CTRI/2019/11/022147.

### 2.4 Sample size

The sample size was calculated for an alpha error of 0.05 and power at 80% based on the proportion of subjects with an effective response at the end of the treatment period. The details of the calculations are given in Supplementary material section.

### 2.5 Study population

#### 2.5.1 Inclusion criteria

The study included male and female participants (30–65 years) with mild to moderate (≤ stage 2) nonalcoholic steatohepatitis (NASH) with or without fibrosis as diagnosed by elevated ALT and Ultrasonography. Normal echotexture of the liver was graded as the absence of steatosis (score 0), a slight and diffuse increase of liver echogenicity with normal visualization of the diaphragm and portal vein as mild (score 1), a moderate increase of liver echogenicity with an impaired appearance of the diaphragm and portal vein as moderate (score 2), and as severe (score 3), when the liver echogenicity showed a marked increase with poor or no visualization of the portal vein wall, diaphragm, and posterior part of the right liver lobe (22). The participants had a body mass index (BMI) in the range of 25–35 kg/m^2^ and had controlled diabetes, hyperlipidemia, or hypertension for at least 3 months before screening. Other inclusion criteria were agreeing to use a barrier method of contraception during the study period for female patients and agreeing to adhere to assessments and visit schedules. Informed consent to participate in the study was signed by all the participants.

#### 2.5.2 Exclusion criteria

Individuals with liver enzymes three times higher than the upper limit of the normal, fatty liver caused by viral hepatitis, autoimmune hepatitis, Wilson disease, any drugs, or alcoholism, as defined by the inability to control drinking due to both physical & emotional dependence on alcohol, were excluded from the study. Patients with biliary tract disease, diabetes (HbA1c >7.2%), dyslipidemia, endocrine diseases, and clinical suspicion of advanced liver disease, cirrhosis, or malignancy were also excluded from the study. Other criteria for exclusion were a history of renal, pulmonary, epileptic, hematologic, cardiovascular, neurological, or psychiatric illness, immunodeficiency diseases, positive HIV test results, and participation in any other clinical trial within the last 3 months.

#### 2.5.3 Randomization

Subjects were randomized using a predetermined block randomization schedule generated using a computer-based randomization software (SAS 9.3), prepared by a statistician, independent of the sponsoring organization, and not involved in the conduct or reporting of the study. The randomization codes were kept strictly confidential and were accessible only to authorized persons on an emergency basis as per the standard operating procedures until the time of unblinding.

#### 2.5.4 Intervention

The study participants were instructed to consume active (GCP- 450 mg tablet) or a matching placebo containing microcrystalline cellulose (450 mg tablet) twice a day after breakfast and dinner for 90 days. Compliance was assessed by recording the number of tablets dispensed to and administered by the subject and returned at each visit in the case record form. All the patients enrolled in the study were asked to initiate lifestyle changes (a healthy diet with cardio/exercise for 5 days a week for at least 30 min) along with placebo or GCP supplements.

### 2.6 Outcome

The primary efficacy endpoint was the mean changes in AST, and ALT enzymes from the screening visit to day 90 and the changes in liver fat content as measured by Fibroscan with CAP from the baseline visit to day 90. The secondary endpoints included changes in liver parameters (total bilirubin, alkaline phosphatase), lipid Profile (LDL, VLDL, HDL, TC, and TG), glycemic Profile (fasting glucose, HbA1c), biomarkers (IL-6, hs-CRP, adiponectin), anthropometric parameters (BMI, abdominal circumference) from baseline to day90 and safety outcome by the incidence of adverse events.

#### 2.6.1 Transient elastography

The transient elastography devices used in this study were Fibroscan 430 Mini Plus (Echosens, France) by trained nurses and physicians. Fibroscan analysis was performed after 3 h of fasting. Patients were placed in a supine position and measurements were done by scanning the liver lobe through an intercostal space. CAP is an average estimate of ultrasound attenuation at 3·5 MHz and is expressed in db/m. LSM by VCTE is an average estimate of stiffness (Young’s modulus) at a shear wave frequency of 50 Hz and is expressed as kPa. Only examinations with at least 10 valid individual measurements were deemed reliable.

#### 2.6.2 Biochemical analysis

Fasting blood samples (10 mL) were collected from each participant, via venipuncture with a Vacutainer system (Becton Dickinson Biosciences, NJ, United States) by trained laboratory staff (EDTA treated tubes for hematology and untreated tubes for biochemistry tests). Hematological indices were measured by Sysmex XN1000 (Sysmex Corporation, Kobe, Japan); and biochemical indices by Cobas 400 (Roche Diagnostics, Mannheim, Germany) per manufacturer’s instructions. Biomarkers IL6 and adiponectin were measured using commercial ELISA kits as per manufacturer’s instructions.

The FibroScan-AST (FAST) score is a noninvasive diagnostic score, which takes the LSM, CAP measurements by Fibroscan, and the AST values into consideration, and provides an efficient method to identify patients at risk of progressive NASH in clinical trials or treatments (23). The FAST scores were calculated from LSM, CAP, and AST values using the equation,


$$\begin{array}{l} \mathrm{FAST}=\{\exp\;(-1.65+1.07\times \ln\left(LSM\right)\\ \;+\;2.66\times 10^{-8}\times {\mathrm{CAP}}^{3}-63.3\times {AST}^{-1})\}\\ \;/\{1+\exp\;(-1.65+1.07\times \ln\left(LSM\right)\\ \;+2.66\times 10^{-8}\times \;{\mathrm{CAP}}^{3}-63.3\times {AST}^{-1})\} \end{array}$$

as described earlier (23). All biochemical parameters were evaluated in a centralized lab with NABH accreditation as per the regulatory requirement (Elbit Diagnostics, Bangalore). Safety was evaluated by laboratory hematological, urinary, and biochemical parameters and by monitoring any incidence of adverse events.

### 2.7 Statistical analysis

All the statistical analysis was carried out by an independent statistician who was blinded to the study. A descriptive univariate analysis of demographic and clinical variables was performed by STATA Software version 16.0. The data gathered was considered as either continuous or categorical variables. All the analysis was conducted on the study population who completed the study. For continuous variables, the normality of distribution was tested by a one-sample Shapiro–Wilk normality test. The quantitative variables were described either as mean and standard deviation or median and interquartile range based on the normality. The primary and secondary efficacy endpoints were compared between the groups by Mann–Whitney U tests. Repeated measures one-way ANOVA followed by Dunnett’s *post-hoc* test were performed for comparing the parameters at every visit with baseline values, within the group to understand the trend in their change. The categorical variables were compared by Chi-square test and the frequency and percentage of the population were presented. The change in quantitative variables from baseline to end of the study was compared to differentiate the treatment effect between the treatment groups. The level of statistical significance is defined as *p* < 0.05.

## 3 Results

### 3.1 Demographic and baseline characteristics

**Figure 3:**
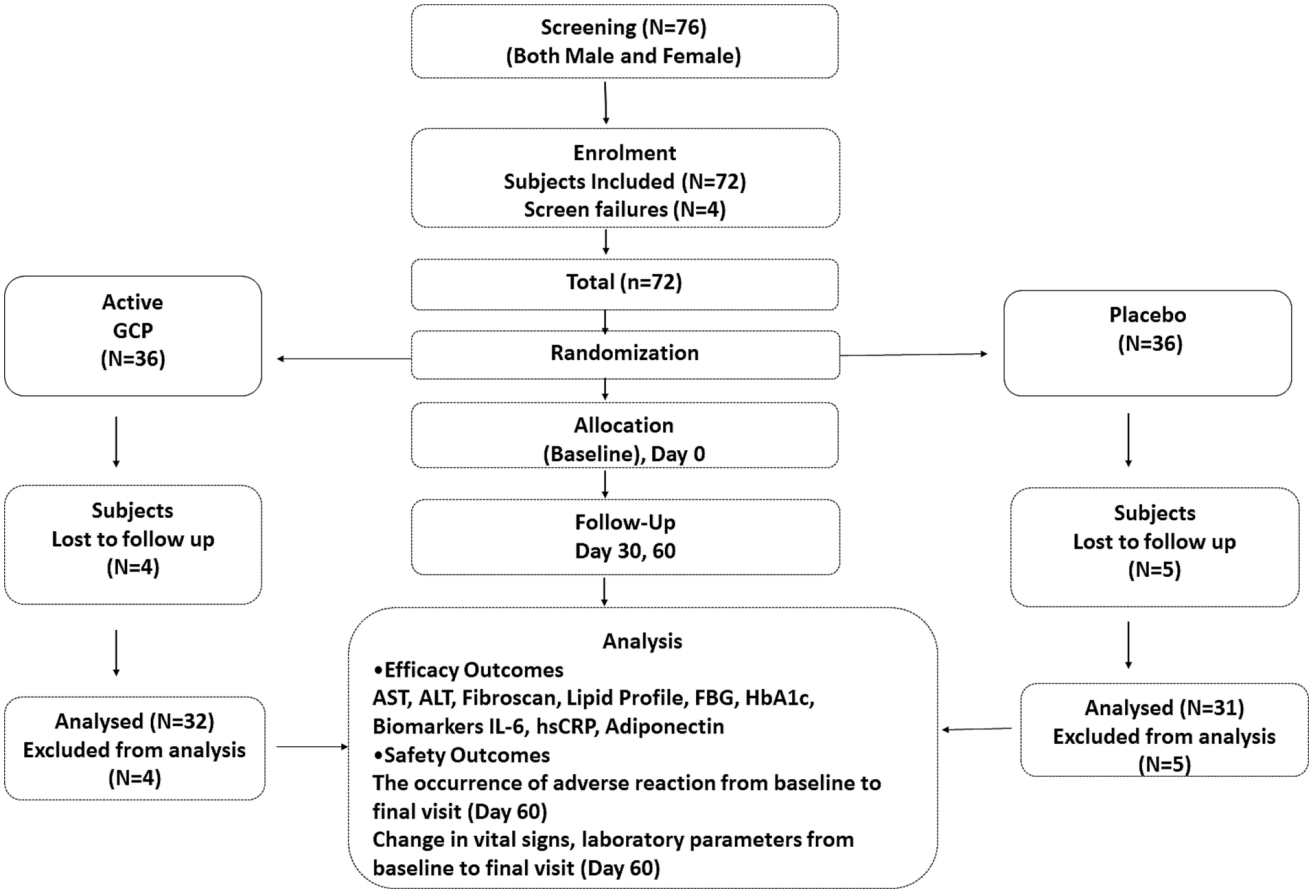
Consort diagram. Consort flow diagram showing the details of enrollment and disposition of participants.

A total of 72 patients were randomized to active and placebo groups at baseline. Nine patients were lost to follow-up, and 63 participants completed the study, 32 in active (14 male and 18 female) and 31 (22 male and 9 female) in the placebo group (Figure 3). The baseline characteristics of the two groups were comparable. All the participants were Asian and had no history of smoking, drug abuse, or alcoholism. The median ALT and AST values were 69.92 ± 1.1 and 61.9 ± 1.7 IU/L, respectively. As per ultrasound analysis, 38.1% (*n* = 24) patients had grade 1 fatty liver, and 61.9% (*N* = 39) had a grade 2 stage of fatty liver. The median LSM value was 11 (7.0–12) kPa, and 79.3% had a score higher than > 7.0 kPa. The median CAp value was 266 (254–282) db/m, and 63.5% of patients showed over 34% fat deposition in the liver (Table 1). Five patients were given metformin at 500 mg/day (four in placebo and one in GCP) during the study. All the patients (both in placebo and GCP groups) enrolled in the study were asked to walk for 30 min, 5 days a week. The instruction for a healthy diet included avoiding fried and processed food and consuming home-cooked food. The patients were not treated with any pharmacological drug for NAFLD during the study period.

**Table 1:** Demographic and baseline characteristics.

Characteristics	PLACEBO (*N* = 31)	GCP (*N* = 32)	Overall (*N* = 63)	*P*-value
Age (years)	48.74 ± 9.1	47.88 ± 8.5	48.30 ± 8.7	0.61
Gender				
Male	22 (70.9%)	14 (43.8%)	36 (57.1%)	0.04
Female	9 (29.03%)	18 (56.3%)	27 (42.9%)	
Height (cm)	167.29 ± 9.5	163.32 ± 9.9	165.27 ± 9.9	0.12
Weight (kg)	83.72 ± 9.6	79.03 ± 9.2	81.34 ± 9.6	0.052
BMI (kg/m^2^)	29.75 ± 2.4	29.79 ± 2.5	29.77 ± 2.4	0.93
WC (cm)	98.40 ± 11.0	97.04 ± 9.5	97.71 ± 10.2	0.50
FBS	111.08 ± 25.1	109.48 ± 15.1	110.28 ± 20.8	0.82
Liver Enzymes (IU/L)				
AST	60.74 ± 14.7	63.09 ± 16.9	61.9 ± 1.7	0.19
ALT	69.16 ± 14.9	70.69 ± 14.3	69.92 ± 1.1	0.27
USG analysis				
Fatty liver grade I	10 (32.3%)	14 (43.8%)	24 (38.1%)	0.43
Fatty liver grade II	21 (67.7%)	18 (56.3%)	39 (61.9%)	
Fibroscan analysis				
CAP (db/m)	267.0 (255, 282)	264.0 (253.3, 286)	266 (254–282)	0.75
LSM (kPa)	11.10 (7.4, 12.0)	11.60 (7.5–12.5)	11 (7.0–12.0)	0.32

The values represent mean ± standard deviation for parametric and median and interquartile values for nonparametric data, except for gender and USG analysis where the number of participants and percentage are given. Continuous variables were compared by the Mann Whitney U test and categorical variables by the Chi-Squared test. WC, waist circumference; Hb1AC, Glycated hemoglobin; ALT, Alanine aminotransferase; AST, Aspartate aminotransferase; USG, Ultrasonography. *p < 0.05 between active and placebo.

### 3.2 Primary endpoints

#### 3.2.1 Change in liver transaminases

The primary efficacy endpoints were the changes in Fibroscan score for liver stiffness and fat accumulation and the change in serum levels of liver transaminases from baseline (day 0) to the end of the study (day 90). The mean reduction in AST in the GCP-supplemented patients from baseline to the end of the study was 9.53 IU/L (15.1%) which was significant (*p* < 0.001) compared to placebo (3.16 IU/L) (Table 2). The mean AST reduced from 60.74 ± 14.7 to 57.58 ± 19.2 in placebo and from 63.09 ± 16.9 to 53.56 ± 17.3, *p* < 0.001 in patients supplemented with GCP for 90 days. Similarly mean ALT values reduced from 69.16 ± 14.9 to 61.73 ± 22.8 in placebo and 70.69 ± 14.3 to 57.22 ± 18.9 in GCP, a mean reduction of 13.5 IU/L (19.1%) in 90 days, significantly (*p* < 0.001) better than placebo (7.4 IU/L, 10.6%) (Table 2).

**Table 2:** The change in liver transaminases, liver fat and stiffness from baseline to day 90 as primary end points.

Parameter	Group	Day 0	Day 90	Mean change D90- D0	P1
AST (IU/L)	Placebo	60.74 ± 14.7	57.58 ± 19.2	−3.16 (−6.78, 0.47)	<0.001
	GCP	63.09 ± 16.9	53.56 ± 17.3*	−9.53 (−12.79, −6.27)	
ALT (IU/L)	Placebo	69.16 ± 14.9	61.73 ± 22.8*	−7.43 (−13.12, −1.75)	0.002
	GCP	70.69 ± 14.3	57.22 ± 18.9*	−13.47 (−18.43, −8.51)	
LSM (kPa)	Placebo	9.80 ± 3.00	9.86 ± 2.56*	0.064 (−0.89, 1.03)	0.001
	GCP	10.23 ± 3.38	9.60 ± 2.87**	−0.56 (−1.4, 0.26)	
CAP (db/m)	Placebo	270.5 ± 22.36	260.1 ± 30.75	−10.42 (−21.59,0.75)	0.03
	GCP	269.6 ± 22.17	257.2 ± 21.42*	−12.38 (−17.37, −7.38)	
FAST Score	Placebo	0.54 ± 0.12	0.49 ± 0.17	−0.04 (−0.088, 0.008)	0.001
	GCP	0.55 ± 0.17	0.46 ± 0.17**	−0.08 (−0.12, −0.04)	

The Mean ± Standard deviation values are represented in the table. The mean change and 95% confidence interval of the mean are given for the difference from day 0. Paired T test was used to compare the changes in the parameters from days 0 to 90 within placebo and GCP. Mann–Whitney U tests was used for the comparison of change in the values from days 0 to 90 between the groups. ALT, Alanine aminotransferase; AST, Aspartate aminotransferase; CAP, Controlled attenuation parameter (fat attenuation by Fibroscan); LSM, Liver stiffness measurement by Fibroscan; FAST, Fibroscan AST score. *P < 0.05, **P < 0.01, from baseline to end of the study within the groups. P1: The significance of the change in the parameter between placebo and GCP, Normal values of AST – 8–48 IU/L and ALT- 7–55 IU/L.

#### 3.2.2 Change in liver fat and fibrosis

The Fibroscan data showed a significant (*p* < 0.001) mean reduction in liver stiffness of 0.57 kPa [10.23 ± 3.38 to 9.66 ± 2.87] in GCP compared to an increase of 0.06 kPa [9.80 ± 3.00 to 9.86 ± 2.56] in placebo. The reduction in liver stiffness was 5.5% in GCP compared to placebo, which was statistically significant (*p* = 0.001). The CAP score decreased by 12.4 db/m in GCP [269.6 ± 22.17 to 257.2 ± 21.42] versus 10.4 db/m in placebo [270.5 ± 22.36 to 260.1 ± 30.75]. The reduction in fat attenuation was 4.6% in GCP compared to 3.8% in placebo (*p* = 0.03). We calculated the FAST score, which identifies patients at risk of progressive NASH (23, 24). The FAST score showed a significant reduction in the GCP group [0.55 ± 0.17 to 0.46 ± 0.17 P,0.001], while it was [0.54 ± 0.12 to 0.49 ± 0.17], accounting for a 16.3% reduction by GCP which was significantly better than 9.2% in placebo (*p* = 0.001) (Table 2).

#### 3.2.3 The trend in the decrease of liver enzymes within the groups

**Figure 4:**
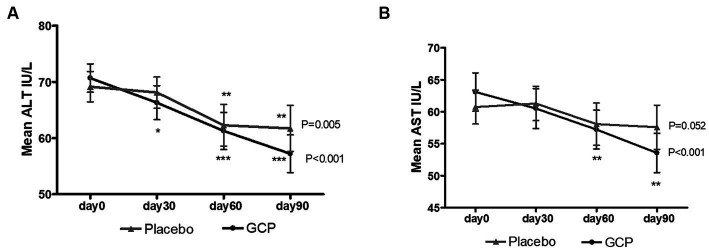
The trend in change in liver transaminases in (A) ALT and (B) AST GCP and Placebo during the study. The Mean ± Standard deviation values are represented for AST and ALT levels. Repeated measure ANOVA (*p-*value) and Dunnett’s post-test (*), with day 0 values as control, were used to compare the changes in AST and ALT within placebo and GCP.

The liver transaminases were monitored every month to understand their change within the group. The decrease in liver enzymes was observed at day 30 and continued up to day 90 in GCP. The placebo group also showed a reduction in ALT and AST, which could be because of exercise, although the effect was lower than that observed in GCP (Figure 4).

#### 3.2.4 Gender and concomitant medicine

Gender-specific analysis retained the effect of GCP in males, while in females, only liver stiffness was significantly better than placebo, which could be due to the lower number of females in the placebo (Supplementary Tables S1A,B). Five participants were taking metformin to control diabetes (4 in placebo and 1 in GCP). Excluding these participants from the analysis did not change the outcome of the study (Supplementary Tables S2A,B).

### 3.3 Secondary endpoints

#### 3.3.1 Lipid profile

Interestingly we observed a reduction in total cholesterol (13.8 vs. 1.96 mg/dL), triglycerides (23.75 vs. 8.59 mg/dL), LDL-C (6.5 vs. 3.6 mg/dL), and VLDL-C (5.1 vs. 1.71 mg/dL) with GCP supplementation in comparison to placebo (Table 3). Repeated measure ANOVA analysis showed a significant trend in total cholesterol, triglycerides, and VLDL-C decrease in GCP (*p* < 0.01), but the trend in LDL-C decrease was not significant (Table 3). HDL-C levels were not affected by GCP and placebo (Table 3).

**Table 3:** Effect of GCP on lipid parameters.

Parameter	Group	Day 0	Day 30	Day 60	Day 90	Repeated measure ANOVA	*P*-value^#^
TC	Placebo	258.19 ± 37.45	248.77 ± 45.1	251.5 ± 42.45	256.23 ± 44.37	0.350	<0.001
	Difference from day 0		−9.42 (−21.88, 3.04)	−6.69 (−20.75, 7.36)	−1.96 (−17.46, 13.52)		
	GCP	261.21 ± 33.98	252.78 ± 37.2*	252.72 ± 31.46*	247.38 ± 27.32*	<0.001	
	Difference from day 0		−8.43 (−13.70, −3.16)	−8.49 (−14.18, −2.81)	−13.83 (−20.92, −6.75)		
TG	Placebo	192.68 ± 42.07	180 ± 45.52	187.57 ± 44.26	184.09 ± 56.20	0.597	<0.001
	Difference from day 0		−12.68 (−30.13, 4.77)	−5.11 (−19.7, 9.47)	−8.59 (−25.91, 8.73)		
	GCP	191.13 ± 40.72	182.34 ± 45.03	172.91 ± 49.19	167.38 ± 25.17	0.008	
	Difference from day 0		−8.79 (−21.65, 4.07)	−18.22 (−32.26,-4.20)	−23.75 (−40.16, −7.35)		
LDL	Placebo	142.26 ± 26.95	132.16 ± 24.36	133.21 ± 20.47	138.6 ± 27.33	0.099	0.001
	Difference from day 0		−10.10 (−20.05,- 0.13)	−9.05 (−20.57, 2.46)	−3.66 (−14.54, 7.22)		
	GCP	143.21 ± 19.97	137.94 ± 21.85	140.13 ± 19.13	136.66 ± 13.13	0.202	
	Difference from day 0		−5.27 (−10.94, 0.40)	−3.08 (−9.68, 3.51)	−6.55 (−13.38, 0.27)		
VLDL	Placebo	43.26 ± 8.85	40.16 ± 10.34	42.09 ± 9.47	41.55 ± 12.76	0.449	0.005
	Difference from day 0		−3.10 (−6.56, 0.37)	−1.17 (−4.10, 1.77)	−1.71 (−5.22, 1.82)		
	GCP	43.75 ± 8.6	41.91 ± 9.81*	39.59 ± 10.66*	38.69 ± 6.20*	0.003	
	Difference from day 0		−1.84 (−4.49, 0.80)	−4.16 (−6.96, −1.35)	−5.06 (−8.47,- 1.65)		
HDL	Placebo	39.29 ± 5.14	38.23 ± 3.87	36.79 ± 3.08	37.35 ± 3.66	0.008	0.050
	Difference from day 0		−1.06 (−2.12, −0.01)	−2.5 (−4.27, −0.72)	−1.94 (−3.43, −0.45)		
	GCP	40.22 ± 3.47	40.0 ± 3.09	39.47 ± 2.49	39.5 ± 2.23	0.215	
	Difference from day 0		−0.22 (−0.88, 0.75)	−0.75 (−1.82, 0.32)	−0.72 (−1.65, 0.21)		

The Mean ± Standard deviation values are represented for lipid levels. The mean change and 95% confidence interval of the mean are given for the difference from day 0. Repeated measure ANOVA and Dunnett’s post-test (*), with day 0 values as control, were used to compare the changes in the parameters within placebo and GCP. Mann–Whitney U tests was used for the comparison of change in the values from day 0 to day 90 between the groups. TC, Total cholesterol; TG, triglycerides; LDL, Low-density lipoprotein cholesterol; HDL, High-density lipoprotein cholesterol. *P < 0.05, **P < 0.01, ***P < 0.001 from baseline to end of the study within the group. ^#^The significance of the change in the parameter between placebo and GCP at day 90. The normal ranges were TG < 150 mg dL^−1^, TC < 200 mg dL^−1^, LDL < 100 mg dL^−1^, VLDL < 30 mg dL^−1^, HDL 40–60 mg dL^−1^.

#### 3.3.2 Body weight and metabolic markers

The mean body weight (2.28 vs. 0.3 kg), BMI (1.18 vs. 0.01), and waist circumference (1.45 vs. 0.35 cm) decreased significantly in GCP-supplemented patients compared to placebo. Body weight decrease was observed in 90.6% of patients in GCP (29/32) compared to 29% (9/31) in placebo. Similarly, waist circumference and BMI also decreased in over 90% of patients in GCP compared to 30% in placebo. Body weight, abdominal circumference, and BMI reductions were significantly better in GCP compared to placebo. Total bilirubin decreased, and we observed a nonsignificant and clinically irrelevant increase in alkaline phosphatase levels in patients supplemented with GCP. Fasting blood sugar showed a decrease in both groups and was not significant in GCP compared to placebo, while HbA1c did not change in both groups (Table 4).

**Table 4:** Effect of GCP on Secondary efficacy outcomes.

Parameter	Group	Day 0	Day 90	Mean change	*P*-value^#^
Body Weight (kg)	Placebo	83.72 ± 9.61	83.42 ± 9.14	−0.3	<0.001
	GCP	79.03 ± 9.25	76.75 ± 8.92**	−2.28	
BMI (kg/m^2^)	Placebo	29.75 ± 2.36	29.74 ± 2.15	0.01	<0.001
	GCP	29.79 ± 2.51	28.61 ± 1.67**	−1.18	
WC (cm)	Placebo	98.4 ± 11.00	98.05 ± 10.60	−0.35	< 0.001
	GCP	97.04 ± 9.46	95.59 ± 8.74**	−1.45	
Bilirubin (mg/dL)	Placebo	0.73 ± 0.26	0.78 ± 0.24	0.05	0.007
	GCP	0.82 ± 0.30	0.73 ± 0.21	−0.08	
ALP (IU/mL)	Placebo	78.29 ± 20.88	83.87 ± 19.52	5.58	0.88
	GCP	83.06 ± 20.17	93.36 ± 30.24	12.44	
FBS (mg/dL)	Placebo	111.09 ± 25.26	105.66 ± 18.29	−5.43	0.172
	GCP	109.51 ± 15.81	98.91 ± 13.21	−10.61	
HbA1c (%)	Placebo	6.42 ± 0.35	6.67 ± 0.14	0.25	0.18
	GCP	6.35 ± 0.49	6.34 ± 0.58	−0.01	

The Mean ± Standard deviation values are represented in the table. Paired *t*-test was used to compare the changes in the parameters from days 0 to 90 within placebo and GCP. Mann–Whitney U tests was used for the comparison of change in the values from days 0 to 90 between the groups. BMI, Body mass index; WC, Waist/Abdominal circumference; ALP, Alkaline phosphatase; FBS, Fasting blood sugar; HbA1c, glycosylated hemoglobin. *P < 0.05, **P < 0.01, ***P < 0.001 from baseline to end of the study within the group. ^#^The significance of the change in the parameter between placebo and GCP at day 90. The normal ranges were ALP- 40 to 129 IU/L, Bilirubin- 0.1 to 1.2 mg dL^−1^ FBS < 100 mg/dL, and HbA1c < 5.7%.

#### 3.3.3 Inflammatory markers

**Figure 5:**
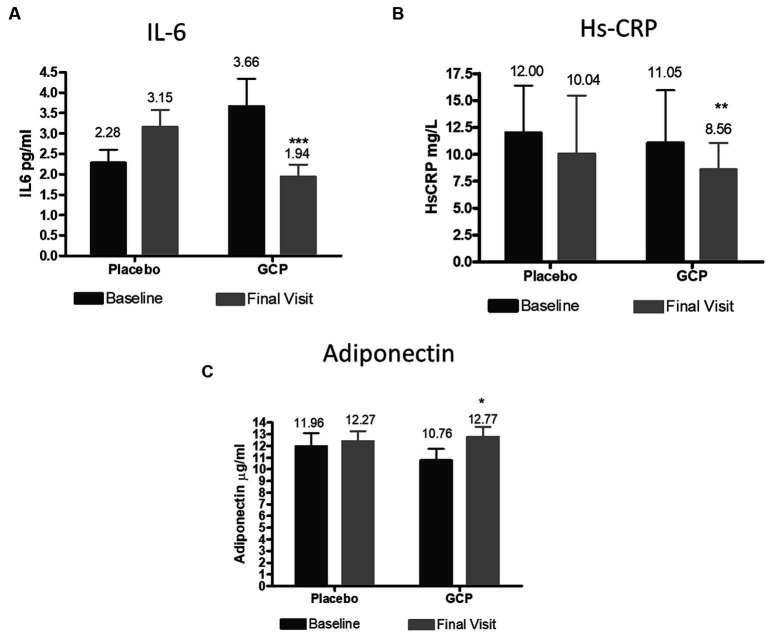
Effect of GCP on inflammatory markers (A) IL 6, (B) HsCRP and (C) Adiponectin. The Mean ± Standard deviation values are represented. The reduction IL6 and Hs-CRP were 0.80 and −1.72 (*p* < 0.001) and −1.8 and − 2.48 (*p* = 0.02) for placebo for GCP, respectively. The increase in adiponectin was 0.42 for placebo and 2.02 for GCP (*p* < 0.001) by Mann–Whitney test. IL6, Interleukin 6; HsCRP, High sensitivity C reactive protein. *p < 0.05, **p < 0.01 and ***p < 0.001 from baseline to end of the study within the group.

The inflammatory cytokine Interleukin-6 and C reactive protein showed a significant decrease by 0.64 pg./mL and 3 mg/L in GCP, which was significant compared to placebo. Adiponectin, the hormone which prevents lipid storage and protects the liver from inflammation and fibrosis, increased from the baseline values by 1.45 μg/mL (13.5%) in GCP, while placebo showed no effect on adiponectin levels (Figure 5).

### 3.4 Safety parameters

Statistical analysis of safety was performed for all the patients who completed the study. Mild adverse events were reported by three participants from the placebo group. The events were resolved without any medication and were not related to the study medication (Table 5). No adverse event was reported in the GCP group. There were no significant differences between the treatment groups for the clinical laboratory examination results. All the laboratory parameters were within the specified normal range (Supplementary Tables S3–S5). These results suggest that Garcinol 20% (50 mg) with Curcuminoids 95% (250 mg) and piperine (5 mg) combination (GCP) is safe for use in NASH without liver cirrhosis patients.

**Table 5:** List of adverse events and their resolution.

Participant no.	Gender- age	Group	Event term	Start date	End date	Outcome
4,013	M-59	Placebo	Diarrhea	12-Sep-21	14-Sep-21	Resolved
4,015	F-64	Placebo	Urinary tract infection	07-Oct-21	09-Oct-21	Resolved
4,016	M-47	Placebo	Lower abdominal pain	08-Oct-21	09-Oct-21	Resolved

Description of mild adverse events experienced by participants during the study. M, male; F, Female.

## 4 Discussion

The present study reports the beneficial effects of a combination of garcinol and curcuminoids with piperine (GCP) supplementation in improving liver parameters, systemic inflammation, and dyslipidemia in patients with mild to moderate NASH. It is probably the first study to document the effect of the combination of garcinol and curcuminoids on liver fibrosis and steatosis assessed using transient elastography (FibroScan).

Currently, no specific pharmacological treatments for NAFLD and NASH are approved by the FDA, although numerous drugs are being evaluated (7). NAFLD is associated with several other comorbidities like diabetes, cardiovascular disease, kidney disease, and cancer and is considered the manifestation of metabolic syndrome in the liver (25, 26). A few promising drugs in phase 3 evaluation are Obetocholic acid -a Farnesoid X or bile acid receptor agonist, Resmetirom, the thyroid hormone receptor beta agonist, Armachol, stearoyl coenzyme A desaturase inhibitor, and Semaglutide, glucagon-like peptide-1 agonist (27, 28). Lifestyle modification through a hypocaloric diet and exercise are the basis for the treatment of NAFLD in the absence of approved pharmacological agents.

Given the complex pathophysiology of NASH, a combination of different drugs with diverse mechanisms of action, therapeutic approaches targeting each stage of the disease may be required to manage the disease progression (29). Herbal and traditional medicine have garnered attention in recent years to effectively manage NAFLD (30). Plant polyphenols were found to have a protective effect in reducing inflammation, oxidative stress, and liver steatosis in animal models (31). In clinical trials, resveratrol supplementation was found to improve inflammatory profile in NAFLD patients, while hesperidin was reported to ameliorate steatosis, glycemia, and hepatic enzymes (32, 33). Flax seeds and Coenzyme Q10 supplementation were reported to reduce inflammation and improve liver function (34, 35). Curcumin is known to interact with multiple molecular targets and alter their gene expression and signaling pathways (36). In animals, curcumin supplementation was shown to reduce obesity and glucose intolerance, fibrosis, and intrahepatic accumulation of CD4^+^ cells in association with NAFLD (37). Clinical trials with curcumin have shown a reduction in triglycerides, blood glucose, and liver enzymes in individuals with metabolic complications (38). Garcinol is an antiadipogenic agent which activates the AMPK pathway and reduces endoplasmic reticulum stress in adipose tissues (13, 14). Garcinol was also shown to modulate gut microbiota and control inflammation by increasing the intestinal commensal bacteria, *Akkermansia* (13).

We earlier reported that the combination of garcinol and curcuminoids reduced NAFLD activity score, fibrosis, and steatosis in a stelic animal model (STAM™) of NASH. The effect of the combination was significantly better than individual treatment with either curcuminoids or garcinol. Curcuminoids were found to reduce inflammation mediated by NF-κB and fibrosis, while garcinol was effective in modulating macrophage activity and reducing steatosis. The combination was effective in targeting both inflammation and steatosis (15). The current clinical study validates the preclinical data of GCP in a clinical setting.

All the study participants, irrespective of the study intervention were asked to walk for 30 min every day for at least 5 days in a week as a lifestyle modification and were not treated with any pharmacological drug. No specific diet was proposed as the participants were from different parts of the country with varied food habits. They were only instructed to avoid processed and fried food during the study. The moderate exercise performed by the participants could have had a positive effect on the liver health. Experimental studies have shown that moderate aerobic and anaerobic exercise for 20–60 min, 4–7 days a week for 6 months to improve liver histology ad reverse liver damage (39, 40). It is also possible that the observed results could be due to the combination of moderate exercise and GCP intervention. However, the participants consuming placebo along with moderate exercise, did not show a substantial change in the liver parameters and the difference between GCP and placebo for the outcome measures was significant.

The elevation of liver enzymes is a marker of hepatocyte injury observed in NAFLD patients. Recent studies suggest that a higher level of ALT may be a consequence and predictor for the development of NASH (41). Curcumin supplemented at a dose of 500 mg to 1,000 mg for 8–12 weeks was reported to reduce AST (−2.7 to −9.2 IU/L) and ALT (−2.9 to 2212-11.3 IU/L) in NAFLD patients in earlier studies (12, 42–44). We observed a reduction of 9.5 IU/L for AST and 13.5 IU/L for ALT with GCP, suggesting that the combination may be better than curcuminoids alone.

For NAFLD progressing to NASH, hepatic fibrosis is the standard endpoint. Although liver biopsy is the accepted diagnosis of fibrosis, the use of transient elastography (Fibroscan) to measure liver stiffness through the estimation of the velocity of propagation of a shear wave through liver tissue is emerging as a valid method for assessing fibrosis (45, 46). Our study demonstrated a reduction of 5.5% in LSM and 4.6% in CAP compared to an increase in LSM and a 3.8% decrease in CAP in placebo in 12 weeks, which could be considered moderate activity. However, all the patients (100%) consuming GCP showed a decrease in LSM, while it was observed in only 13% of patients in the placebo group (4/31). It is worthwhile to note that in an earlier clinical study with Saroglitazar, a dual peroxisome proliferator-activated receptor α/γ agonist approved for diabetic dyslipidemia, the reported reduction in LSM was 10.5% after 24 weeks, although the improvement in CAP was much superior (23.5%) (47). In a 10-year study to monitor fibrosis progression in NAFLD patients, an improvement was defined as a reduction in the LSM by more than 2 kPa in 10 years. ALT improvement by more than 30% and more than 1 unit change in BMI (kg/m^2^) was found to contribute to LSM improvement (48).

Several non-invasive methods have been developed to diagnose NASH patients in recent years. The FAST score, which takes the LSM, CAP measurements by Fibroscan, and the AST values into consideration, provides an efficient non-invasive method to identify patients at risk of progressive NASH for clinical trials or treatments (23). The FAST score showed a significant decrease in the GCP group compared to the placebo, further reiterating that the herbal combination can potentially improve overall liver function. These results agree with the preclinical data in the mouse model, where we observed a reduction in fibrosis and steatosis by liver histopathology.

The prognosis of NASH depends on obesity, insulin resistance, and type 2 diabetes as risk factors, and addressing these conditions has been the focus of treatment. Weight loss by bariatric surgery has shown benefits in improving NAFLD (49). The American Association for the Study of Liver Diseases (AASLD) NAFLD practice guidelines also recommend a reduction in body weight by 3–5% to improve hepatic steatosis, while (≥ 7%) reduction to improve fibrosis (50, 51). We observed a 3.8% reduction in weight, 1.8% in waist circumference, and 3.3% in BMI in the GCP group, which suggests an improvement in anthropometric parameters which could have a positive effect on NAFLD. Supplementation for a longer time might improve these parameters further. Cardiometabolic risks are higher in NASH patients, so control of dyslipidemia is another essential factor. Further, excess accumulation of fatty acids in the liver can cause lipotoxicity and hepatocyte injury (52). GCP was helpful in reducing TC (5.3%), TG (12.8%), LDL (4.6%), and VLDL (11.5%) levels while HDL was not affected. Although these are only minimal effects, cumulatively positive effects on multiple parameters would synergistically and effectively improve liver health.

Adequate control of diabetes is critical for NASH since hyperglycemia and insulin resistance contribute to hepatic steatosis. Although we observed some reduction in FBS, no effect was seen with HbA1c, suggesting a minimal effect of GCP supplementation in diabetic control. Several studies have shown a positive effect of curcumin on glycemic control in type 2 diabetic patients (53). Most of these studies used higher doses of curcumin for a longer duration compared to that used in GCP, which could explain the results observed in our study.

Prolonged lipotoxicity causes oxidative stress and ER stress culminating in the activation of toll-like receptors, inflammasomes, and transcription factors involved in the expression of inflammatory markers (51). Leptin and adiponectin secreted from the adipose tissue have a pro and anti-inflammatory effect on liver inflammation. Adiponectin reduces hepatic inflammation, and lipid accumulation and regulates oxidative stress and Kupffer cell polarization (54). A systematic review and meta-analysis of randomized clinical trials reported that curcumin could significantly improve adiponectin concentrations compared to placebo (55). Our results agree with this observation, although the curcumin was used at 1500 mg dose in RCTs compared to 250 mg in our study. An increase in adiponectin was also observed in our preclinical study with the same combination (15).

GCP supplement resulted in a significant reduction in inflammatory markers (IL6 and HsCRP) in the serum, suggesting an improvement in the inflammatory status in these patients. IL6 has been identified as one of the independent prognostic factors for liver steatosis in obese individuals (56). The cytokine has also been observed as a predictive biomarker for the development of NASH in NAFLD patients (57). Thus, a reduction in IL6 may be a significant observation in this study. In an earlier study, Saadati et al. observed a reduction in inflammatory markers in NAFLD patients supplemented with 1,500 mg curcumin for 12 weeks but the changes were not significantly different from placebo (58). Curcuminoids with piperine at 500 + 5 mg was reported to reduce the serum levels of inflammatory markers (TNF-α, MCP1) in another trial (11). Our results suggest that by combining curcumin with garcinol, the effective dose of curcumin could be reduced to get better benefit on inflammatory parameters.

Finally, we believe that the mechanism of action of GCP may be mediated through its action of oxidative stress, inflammation, and energy balance. Fatty liver is a consequence of increased free fatty acids imported into liver from adipocytes, diet and *de novo* hepatic lipogenesis. Earlier studies have shown that garcinol could reduce adipogenesis by reducing ER stress and activating AMPK, the cellular energy sensor. The progression to NASH is facilitated by an elevation of intracellular reactive oxygen species (ROS) that increase oxidative stress inflammation and cell death. Both Curcuminoids and Garcinol have antioxidant properties, with evidence of liver protection against damage induced by alcohol, drugs, and other agents (59–61). Curcuminoids are well studied for their anti-inflammatory activity and health benefits related to chronic inflammatory diseases. Thus, the beneficial effect observed in the present study could be due to the combined effect of reduction in oxidative stress, inflammation, and energy balance by GCP. Further, Garcinol has been observed to prevent gut dysbiosis which is one of the contributing factors for NAFLD (62). Since NAFLD progressing to NASH is a complex disease driven by multiple pathways, the combination of GCP may target some of these pathways at different levels resulting in a better outcome.

All the serum biochemical markers and vital signs were in the normal range throughout the study, and no serious adverse events were reported by any patients during the 90 days of supplementation, suggesting the safety of GCP formulation. The Curcumin C3 complex has been evaluated in more than 100 clinical studies and was found to be safe for consumption at a dose as high as 8 g per day. Garcinol at 40% was also proven to be safe in preclinical studies (63). The combination described in the present study contained much lower doses of curcumin c3 complex and Garcinol and hence could be regarded as a safe composition.

A few limitations, as well as strengths of our study, are worth mentioning. The study was conducted on mild to moderate NASH patients with mild diabetes or dyslipidemia in a relatively small population. We cannot generalize the results for NASH patients with other comorbidities. We did not conduct a liver biopsy to confirm our results which could be another limitation. However, we used validated non-invasive testing using FibroScan, which could be a study strength. The study was conducted in two sites in India, one in the south and the other in the eastern part of the country, which includes a diverse population and could be termed as a strength of the study. The gender distribution in the two groups was not equal, which is also a limitation and future studies need to be conducted in larger population in both male and females. The age distribution was comparable between the two groups and hence a specific age-related analysis was not carried out. The results observed were significant, but the percentage reductions were moderate, and the liver enzymes were not reduced to normal levels. The effects of dietary supplements could not be compared to pharmaceutical drugs, as many of them were conducted over a six-month duration compared to the three-month study detailed here. The study used food components as actives and was found to be safe as reported herein. The study had a gender difference in active and placebo, which could have affected the analysis and is a potential limitation of the study. In the future, we plan to conduct studies on a larger population of NASH patients of different age-groups, for a longer period of time. The development of NASH involves complex mechanisms and its resolution in terms of improvement in liver and serum biomarkers is also a slow process. A study duration of 6–12 months may help to understand the effect of GCP on liver function and other metabolic parameters. Further, GCP may also be combined with other known supplements, like Vitamin E, to optimize its effect in future clinical studies.

## 5 Conclusion

GCP as a dietary supplement with safe food ingredients was effective in improving liver enzymes, liver steatosis and fibrosis, inflammation, and lipid markers in NASH patients. Curcumin has been reported to have favorable effects on NAFLD, at high doses. By combining it with garcinol we observed positive effects at a much lower dose. Thus, GCP could be a potential option as an adjunct supplement along with lifestyle management and other standard treatments for the management of NAFLD progressing to NASH. Future studies for a longer duration in comparison to standard treatment would benefit the clinical use of GCP as an adjunct supplement for the management of NASH.

## Data availability statement

The original contributions presented in the study are included in the article/Supplementary material, further inquiries can be directed to the corresponding author.

## Ethics statement

All study documents were reviewed and the Study Protocol Number CPL/79/G&C_NASH/JUN/19 was approved by the Institutional Ethics committee of MS Ramaiah Medical College and Hospital, 24 December 2019, Dr. Satyprakash’s Center for Digestive and liver diseases, 11 October 2019, College of Medicine and JNM Hospital, 22 June 2020 and Santosh Hospital, 21 January 2021. The studies were conducted in accordance with the local legislation and institutional requirements. The participants provided their written informed consent to participate in this study.

## Author contributions

MM: conceptualization and funding. KN: study design, review. MN: review. SP: clinical study coordination and data curation. CB and SS: principal investigator. LM: study design, data analysis, and writing. All authors contributed to the article and approved the submitted version.

## References

[1] Noureddin M Vipani A Bresee C Todo T Kim IK Alkhouri N. NASH leading cause of liver transplant in women: updated analysis of indications for liver transplant and ethnic and gender variances. Am J Gastroenterol. (2018) 113:1649–59. doi: 10.1038/s41395-018-0088-6 29880964 PMC9083888

[2] Marchesini G Bugianesi E Forlani G Cerrelli F Lenzi M Manini R. Nonalcoholic fatty liver, steatohepatitis, and the metabolic syndrome. Hepatology. (2003) 37:917–23. doi: 10.1053/jhep.2003.50161 12668987

[3] Bellentani S. The epidemiology of non-alcoholic fatty liver disease. Liver Int. (2017) 37:81–4. doi: 10.1111/liv.13299 28052624

[4] Roberts EA. Pediatric nonalcoholic fatty liver disease (NAFLD): a “growing” problem? J Hepatol. (2007) 46:1133–42. doi: 10.1016/j.jhep.2007.03.003 17445934

[5] Younossi ZM Golabi P Paik JM Henry A Van Dongen C Henry L. The global epidemiology of nonalcoholic fatty liver disease (NAFLD) and nonalcoholic steatohepatitis (NASH): a systematic review. Hepatology. (2023) 77:1335–47. doi: 10.1097/HEP.0000000000000004 36626630 PMC10026948

[6] Cotter TG Rinella M. Nonalcoholic fatty liver disease 2020: the state of the disease. Gastroenterology. (2020) 158:1851–64. doi: 10.1053/j.gastro.2020.01.052 32061595

[7] Noureddin M Muthiah MD Sanyal AJ. Drug discovery and treatment paradigms in nonalcoholic steatohepatitis. Endocrinol Diabetes Metab. (2019) 3:e00105–5. doi: 10.1002/edm2.105 33102791 PMC7576222

[8] Vuppalanchi R Noureddin M Alkhouri N Sanyal AJ. Therapeutic pipeline in nonalcoholic steatohepatitis. Nat Rev Gastroenterol Hepatol. (2021) 18:373–92. doi: 10.1038/s41575-020-00408-y, PMID: 33568794

[9] Ngu MH Norhayati MN Rosnani Z Zulkifli MM. Curcumin as adjuvant treatment in patients with non-alcoholic fatty liver (NAFLD) disease: a systematic review and meta-analysis. Complement Ther Med. (2022) 68:102843. doi: 10.1016/j.ctim.2022.102843, PMID: 35661765

[10] White CM Lee JY. The impact of turmeric or its curcumin extract on nonalcoholic fatty liver disease: a systematic review of clinical trials. Pharm Pract. (2019) 17:1350. doi: 10.18549/PharmPract.2019.1.1350, PMID: 31015871 PMC6463416

[11] Saberi-Karimian M Keshvari M Ghayour-Mobarhan M Salehizadeh L Rahmani S Behnam B. Effects of curcuminoids on inflammatory status in patients with non-alcoholic fatty liver disease: a randomized controlled trial. Complement Ther Med. (2020) 49:102322. doi: 10.1016/j.ctim.2020.102322, PMID: 32147075

[12] Panahi Y Valizadegan G Ahamdi N Ganjali S Majeed M Sahebkar A. Curcuminoids plus piperine improve nonalcoholic fatty liver disease: a clinical trial. J Cell Biochem. (2019) 120:15989–96. doi: 10.1002/jcb.28877 31168845

[13] Lee PS Teng CY Kalyanam N Ho CT Pan MH. Garcinol reduces obesity in high-fat-diet-fed mice by modulating gut microbiota composition. Mol Nutr Food Res. (2019) 63:e1800390. doi: 10.1002/mnfr.201970003 30516329

[14] Majeed M Majeed S Nagabhushanam K Lawrence L Mundkur L. *Garcinia indica* extract standardized for 20% Garcinol reduces adipogenesis and high fat diet-induced obesity in mice by alleviating endoplasmic reticulum stress. J Funct Foods. (2020) 67:103863. doi: 10.1016/j.jff.2020.103863

[15] Majeed M Majeed S Nagabhushanam K Lawrence L Mundkur L. Novel combinatorial regimen of garcinol and curcuminoids for non-alcoholic steatohepatitis (NASH) in mice. Sci Rep. (2020) 10:7440. doi: 10.1038/s41598-020-64293-w 32366854 PMC7198554

[16] Shoba G Joy D Joseph T Majeed M Rajendran R Srinivas PS. Influence of piperine on the pharmacokinetics of curcumin in animals and human volunteers. Planta Med. (1998) 64:353–6. doi: 10.1055/s-2006-957450, PMID: 9619120

[17] Badmaev V Majeed M Prakash L. Piperine derived from black pepper increases the plasma levels of coenzyme Q10 following oral supplementation. J Nutr Biochem. (2000) 11:109–13. doi: 10.1016/S0955-2863(99)00074-1, PMID: 10715596

[18] Barve K Ruparel K. Effect of bioenhancers on amoxicillin bioavailability. ADMET DMPK. (2015) 3:45–50. doi: 10.5599/admet.3.1.161

[19] Boddupalli BM Anisetti RN Ramani R Malothu N. Enhanced pharmacokinetics of omeprazole when formulated as gastroretentive microspheres along with piperine. Asian Pac J Trop Dis. (2014) 4:S129–33. doi: 10.1016/S2222-1808(14)60427-8

[20] Johnson JJ Nihal M Siddiqui IA Scarlett CO Bailey HH Mukhtar H. Enhancing the bioavailability of resveratrol by combining it with piperine. Mol Nutr Food Res. (2011) 55:1169–76. doi: 10.1002/mnfr.201100117, PMID: 21714124 PMC3295233

[21] Zhao-Hui J Wen Q Hui L Jiang X-H Ling W. Enhancement of oral bioavailability and immune response of Ginsenoside Rh2 by co-administration with piperine. Chin J Nat Med. (2018) 16:143–9. doi: 10.1016/S1875-5364(18)30041-4 29455730

[22] Hernaez R Lazo M Bonekamp S Kamel I Brancati FL Guallar E. Diagnostic accuracy and reliability of ultrasonography for the detection of fatty liver: a meta-analysis. Hepatology. (2011) 54:1082–90. doi: 10.1002/hep.24452, PMID: 21618575 PMC4197002

[23] Newsome PN Sasso M Deeks JJ Paredes A Boursier J Chan W-K. FibroScan-AST (FAST) score for the non-invasive identification of patients with non-alcoholic steatohepatitis with significant activity and fibrosis: a prospective derivation and global validation study. Lancet Gastroenterol Hepatol. (2020) 5:362–73. doi: 10.1016/S2468-1253(19)30383-8, PMID: 32027858 PMC7066580

[24] Noureddin N Alkhouri N Brown KA Noureddin M. Driving nonalcoholic steatohepatitis forward using the FibroScan aspartate aminotransferase score, but obey the traffic lights. Hepatology. (2020) 72:2228–30. doi: 10.1002/hep.31498 32757393

[25] Angulo P Kleiner DE Dam-Larsen S Adams LA Bjornsson ES Charatcharoenwitthaya P. Liver fibrosis, but no other histologic features, is associated with long-term outcomes of patients with nonalcoholic fatty liver disease. Gastroenterology. (2015) 149:389–397.e10. doi: 10.1053/j.gastro.2015.04.043, PMID: 25935633 PMC4516664

[26] Truong E Noureddin M. The interplay between nonalcoholic fatty liver disease and kidney disease. Clin Liver Dis. (2022) 26:213–27. doi: 10.1016/j.cld.2022.01.008, PMID: 35487606

[27] Majumdar A Verbeek J Tsochatzis EA. Non-alcoholic fatty liver disease: current therapeutic options. Curr Opin Pharmacol. (2021) 61:98–105. doi: 10.1016/j.coph.2021.09.007 34688168

[28] Rinella ME Dufour JF Anstee QM Goodman Z Younossi Z Harrison SA. Non-invasive evaluation of response to obeticholic acid in patients with NASH: results from the REGENERATE study. J Hepatol. (2022) 76:536–48. doi: 10.1016/j.jhep.2021.10.029, PMID: 34793868

[29] Dufour J-F Caussy C Loomba R. Combination therapy for non-alcoholic steatohepatitis: rationale, opportunities and challenges. Gut. (2020) 69:1877–84. doi: 10.1136/gutjnl-2019-319104, PMID: 32381514 PMC7497577

[30] Yan T Yan N Wang P Xia Y Hao H Wang G. Herbal drug discovery for the treatment of nonalcoholic fatty liver disease. Acta Pharm Sin B. (2020) 10:3–18. doi: 10.1016/j.apsb.2019.11.017, PMID: 31993304 PMC6977016

[31] Simón J Casado-Andrés M Goikoetxea-Usandizaga N Serrano-Maciá M Martínez-Chantar ML. Nutraceutical properties of polyphenols against liver diseases. Nutrients. (2020) 12:3517. doi: 10.3390/nu12113517, PMID: 33203174 PMC7697723

[32] Cheraghpour M Imani H Ommi S Alavian SM Karimi-Shahrbabak E Hedayati M. Hesperidin improves hepatic steatosis, hepatic enzymes, and metabolic and inflammatory parameters in patients with nonalcoholic fatty liver disease: a randomized, placebo-controlled, double-blind clinical trial. Phytother Res. (2019) 33:2118–25. doi: 10.1002/ptr.6406, PMID: 31264313

[33] Faghihzadeh F Adibi P Rafiei R Hekmatdoost A. Resveratrol supplementation improves inflammatory biomarkers in patients with nonalcoholic fatty liver disease. Nutr Res. (2014) 34:837–43. doi: 10.1016/j.nutres.2014.09.005, PMID: 25311610

[34] Farsi F Mohammadshahi M Alavinejad P Rezazadeh A Zarei M Engali KA. Functions of coenzyme Q10 supplementation on liver enzymes, markers of systemic inflammation, and Adipokines in patients affected by nonalcoholic fatty liver disease: a double-blind, placebo-controlled, randomized clinical trial. J Am Coll Nutr. (2016) 35:346–53. doi: 10.1080/07315724.2015.1021057, PMID: 26156412

[35] Yari Z Rahimlou M Eslamparast T Ebrahimi-Daryani N Poustchi H Hekmatdoost A. Flaxseed supplementation in non-alcoholic fatty liver disease: a pilot randomized, open labeled, controlled study. Int J Food Sci Nutr. (2016) 67:461–9. doi: 10.3109/09637486.2016.1161011, PMID: 26983396

[36] Shishodia S. Molecular mechanisms of curcumin action: gene expression. Biofactors. (2013) 39:37–55. doi: 10.1002/biof.1041 22996381

[37] Inzaugarat ME De Matteo E Baz P Lucero D García CC Gonzalez Ballerga E. New evidence for the therapeutic potential of curcumin to treat nonalcoholic fatty liver disease in humans. PLoS One. (2017) 12:e0172900–14. doi: 10.1371/journal.pone.0172900, PMID: 28257515 PMC5336246

[38] Mokgalaboni K Ntamo Y Ziqubu K Nyambuya TM Nkambule BB Mazibuko-Mbeje SE. Curcumin supplementation improves biomarkers of oxidative stress and inflammation in conditions of obesity, type 2 diabetes and NAFLD: updating the status of clinical evidence. Food Funct. (2021) 12:12235–49. doi: 10.1039/D1FO02696H, PMID: 34847213

[39] Cigrovski Berkovic M Bilic-Curcic I Mrzljak A Cigrovski V. NAFLD and physical exercise: ready, steady, go! Front Nutr. (2021) 8:734859. doi: 10.3389/fnut.2021.734859, PMID: 34676233 PMC8523679

[40] Eckard C Cole R Lockwood J Torres DM Williams CD Shaw JC. Prospective histopathologic evaluation of lifestyle modification in nonalcoholic fatty liver disease: a randomized trial. Ther Adv Gastroenterol. (2013) 6:249–59. doi: 10.1177/1756283X13484078, PMID: 23814606 PMC3667474

[41] Schindhelm RK Diamant M Dekker JM Tushuizen ME Teerlink T Heine RJ. Alanine aminotransferase as a marker of non-alcoholic fatty liver disease in relation to type 2 diabetes mellitus and cardiovascular disease. Diabetes Metab Res Rev. (2006) 22:437–43. doi: 10.1002/dmrr.666, PMID: 16832839

[42] Navekar R Rafraf M Ghaffari A Asghari-Jafarabadi M Khoshbaten M. Turmeric supplementation improves serum glucose indices and leptin levels in patients with nonalcoholic fatty liver diseases. J Am Coll Nutr. (2017) 36:261–7. doi: 10.1080/07315724.2016.1267597, PMID: 28443702

[43] Panahi Y Kianpour P Mohtashami R Soflaei SS Sahebkar A. Efficacy of phospholipidated curcumin in nonalcoholic fatty liver disease: a clinical study. J Asian Nat Prod Res. (2019) 21:798–805. doi: 10.1080/10286020.2018.1505873, PMID: 30415581

[44] Rahmani S Asgary S Askari G Keshvari M Hatamipour M Feizi A. Treatment of non-alcoholic fatty liver disease with curcumin: a randomized placebo-controlled trial. Phytother Res. (2016) 30:1540–8. doi: 10.1002/ptr.5659 27270872

[45] Eddowes PJ Sasso M Allison M Tsochatzis E Anstee QM Sheridan D. Accuracy of FibroScan controlled attenuation parameter and liver stiffness measurement in assessing steatosis and fibrosis in patients with nonalcoholic fatty liver disease. Gastroenterology. (2019) 156:1717–30. doi: 10.1053/j.gastro.2019.01.042, PMID: 30689971

[46] Wong VW Vergniol J Wong GL Foucher J Chan HL Le Bail B. Diagnosis of fibrosis and cirrhosis using liver stiffness measurement in nonalcoholic fatty liver disease. Hepatology. (2010) 51:454–62. doi: 10.1002/hep.23312 20101745

[47] Goyal O Nohria S Goyal P Kaur J Sharma S Sood A. Saroglitazar in patients with non-alcoholic fatty liver disease and diabetic dyslipidemia: a prospective, observational, real world study. Sci Rep. (2020) 10:21117. doi: 10.1038/s41598-020-78342-x, PMID: 33273703 PMC7713236

[48] Nogami A Yoneda M Kobayashi T Kessoku T Honda Y Ogawa Y. Assessment of 10-year changes in liver stiffness using vibration-controlled transient elastography in non-alcoholic fatty liver disease. Hepatol Res. (2019) 49:872–80. doi: 10.1111/hepr.13349 30974498

[49] Bower G Toma T Harling L Jiao LR Efthimiou E Darzi A. Bariatric surgery and non-alcoholic fatty liver disease: a systematic review of liver biochemistry and histology. Obes Surg. (2015) 25:2280–9. doi: 10.1007/s11695-015-1691-x 25917981

[50] Chalasani N Younossi Z Lavine JE Charlton M Cusi K Rinella M. The diagnosis and management of nonalcoholic fatty liver disease: practice guidance from the American Association for the Study of Liver Diseases. Hepatology. (2018) 67:328–57. doi: 10.1002/hep.29367, PMID: 28714183

[51] Romero-Gómez M Zelber-Sagi S Trenell M. Treatment of NAFLD with diet, physical activity and exercise. J Hepatol. (2017) 67:829–46. doi: 10.1016/j.jhep.2017.05.016 28545937

[52] Neuschwander-Tetri BA. Hepatic lipotoxicity and the pathogenesis of nonalcoholic steatohepatitis: the central role of nontriglyceride fatty acid metabolites. Hepatology. (2010) 52:774–88. doi: 10.1002/hep.23719, PMID: 20683968

[53] Rivera-Mancía S Trujillo J Chaverri JP. Utility of curcumin for the treatment of diabetes mellitus: evidence from preclinical and clinical studies. J Nutr Intermed Metab. (2018) 14:29–41. doi: 10.1016/j.jnim.2018.05.001

[54] Fukushima J Kamada Y Matsumoto H Yoshida Y Ezaki H Takemura T. Adiponectin prevents progression of steatohepatitis in mice by regulating oxidative stress and Kupffer cell phenotype polarization. Hepatol Res. (2009) 39:724–38. doi: 10.1111/j.1872-034X.2009.00509.x, PMID: 19473437

[55] Clark CCT Ghaedi E Arab A Pourmasoumi M Hadi A. The effect of curcumin supplementation on circulating adiponectin: a systematic review and meta-analysis of randomized controlled trials. Diabetes Metab Syndr. (2019) 13:2819–25. doi: 10.1016/j.dsx.2019.07.045, PMID: 31425942

[56] García-Galiano D Sánchez-Garrido MA Espejo I Montero JL Costán G Marchal T. IL-6 and IGF-1 are independent prognostic factors of liver steatosis and non-alcoholic steatohepatitis in morbidly obese patients. Obes Surg. (2007) 17:493–503. doi: 10.1007/s11695-007-9087-1, PMID: 17608262

[57] Mohamed KA Mohamed EE Ahmed DM Sayed MA Hussien AR. A study of interleukin 6 as a predictive biomarker for development of nonalcholic steatohepatitis in patients with Nonalcholic fatty liver disease. QJM Int J Med. (2020) 113:hcaa052.048. doi: 10.1093/qjmed/hcaa052.048

[58] Saadati S Sadeghi A Mansour A Yari Z Poustchi H Hedayati M. Curcumin and inflammation in non-alcoholic fatty liver disease: a randomized, placebo controlled clinical trial. BMC Gastroenterol. (2019) 19:133. doi: 10.1186/s12876-019-1055-4, PMID: 31345163 PMC6659284

[59] Hung WL Tsai ML Sun PP Tsai CY Yang CC Ho CT. Protective effects of garcinol on dimethylnitrosamine-induced liver fibrosis in rats. Food Funct. (2014) 5:2883–91. doi: 10.1039/C4FO00342J, PMID: 25183344

[60] Lee HY Kim SW Lee GH Choi MK Chung HW Lee YC. Curcumin and *Curcuma longa* L. extract ameliorate lipid accumulation through the regulation of the endoplasmic reticulum redox and ER stress. Sci Rep. (2017) 7:6513. doi: 10.1038/s41598-017-06872-y 28747775 PMC5529367

[61] Rivera-Espinoza Y Muriel P. Pharmacological actions of curcumin in liver diseases or damage. Liver Int. (2009) 29:1457–66. doi: 10.1111/j.1478-3231.2009.02086.x 19811613

[62] Leung C Rivera L Furness JB Angus PW. The role of the gut microbiota in NAFLD. Nat Rev Gastroenterol Hepatol. (2016) 13:412–25. doi: 10.1038/nrgastro.2016.85 27273168

[63] Majeed M Bani S Bhat B Pandey A Mundkur L Neupane P. Safety profile of 40% Garcinol from *Garcinia indica* in experimental rodents. Toxicol Rep. (2018) 5:750–8. doi: 10.1016/j.toxrep.2018.06.009, PMID: 29984188 PMC6031240

